# The SDBC is active in quenching oxidative conditions and bridges the cell envelope layers in *Deinococcus radiodurans*

**DOI:** 10.1016/j.jbc.2022.102784

**Published:** 2022-12-09

**Authors:** Domenica Farci, André T. Graça, Luca Iesu, Daniele de Sanctis, Dario Piano

**Affiliations:** 1 Department of Plant Physiology, Warsaw University of Life Sciences - SGGW, Warsaw, Poland; 2 Department of Chemistry, Umeå University, Umeå, Sweden; 3 Department of Life and Environmental Sciences, Laboratory of Plant Physiology and Photobiology, University of Cagliari, Cagliari, Italy; 4 Structural Biology group, ESRF, The European Synchrotron Radiation Facility, Grenoble, France

**Keywords:** cell envelope, SDBC, S-layer, porins, SlpA, DR_2577, DR_0644, superoxide dismutase, Cu-only SOD, ROS, reactive oxygen species

## Abstract

*Dein*ococcus radiodurans is known for its remarkable ability to withstand harsh stressful conditions. The outermost layer of its cell envelope is a proteinaceous coat, the S-layer, essential for resistance to and interactions with the environment. The S-layer Deinoxanthin-binding complex (SDBC), one of the main units of the characteristic multilayered cell envelope of this bacterium, protects against environmental stressors and allows exchanges with the environment. So far, specific regions of this complex, the collar and the stalk, remained unassigned. Here, these regions are resolved by cryo-EM and locally refined. The resulting 3D map shows that the collar region of this multiprotein complex is a trimer of the protein DR_0644, a Cu-only superoxide dismutase (SOD) identified here to be efficient in quenching reactive oxygen species. The same data also showed that the stalk region consists of a coiled coil that extends into the cell envelope for ∼280 Å, reaching the inner membrane. Finally, the orientation and localization of the complex are defined by in situ cryo-electron crystallography. The structural organization of the SDBC couples fundamental UV antenna properties with the presence of a Cu-only SOD, showing here coexisting photoprotective and chemoprotective functions. These features suggests how the SDBC and similar protein complexes, might have played a primary role as evolutive templates for the origin of photoautotrophic processes by combining primary protective needs with more independent energetic strategies.

Studying the resistance mechanisms of extremophiles provides a preferential view for understanding the encrypted processes aimed at preventing and repairing oxidative stress in biology. This is due to the fact that, in extremophiles, stressors intensities and mechanisms to cope with their effects are expected to be more pronounced with respect to other organisms (*e.g.*, mesophiles); hence, their resistance mechanisms are easier to study. The bacterium *Deinococcus radiodurans* is one of the most intriguing organisms for what concerns the resistance under extreme conditions, having an amazingly high resistance to electromagnetic stress and especially to gamma rays, X-rays, and UV light. This organism couples an efficient repair system, which includes a high turnover of its genome, transcriptome, and proteome (1, 2) with an equally efficient damage prevention system. In this respect, photoprotective processes, which are taking place on the cell envelope of this bacterium (3, 4), are coordinated with chemoprotective reactions aimed at quenching the reactive oxygen species (ROS) induced in the cytosol (5, 6, 7). Here, we report structural and functional evidence that indicates the main cell envelope/surface layer (S-layer) complex of *D. radiodurans* as an assembly that is able to prevent damage by photoprotective and chemoprotective mechanisms.

The S-layer is an external layer of the cell envelope that surrounds the whole cell body of many prokaryotes and functions as an external barrier facing the environment (8, 9). This proteinaceous assembly has undefined properties that span from the protection against environmental stressors (*e.g.*, radiation, heat, desiccation, pressure) to the host invasion in case of some pathogenic species (10, 11, 12, 13). These structures are fine supramolecular organizations of regularly repeated monoprotein or multiprotein units, resulting in fascinating patterns with crystalline regularity. Because of their self-assembling properties and the presence of extended hydrophobic regions, S-layer proteins are difficult to handle and only a few S-layers could be studied in molecular detail (14, 15). For these reasons, the S-layers' functionality and ecophysiology are still poorly understood.

The cell envelope/S-layer of the radiation-resistant bacterium *D. radiodurans* is one of the most characterized (16, 17, 18, 19, 20, 21). Several studies described the overall organization of this system (19, 20, 21, 22), also providing biochemical and functional details of its protein components (3, 4, 12, 23, 24). The crystalline cell envelope/S-layer of *D. radiodurans* is made by three different multisubunit protein complexes (19, 20) among which the main one is the S-layer Deinoxanthin-binding complex (SDBC), composed of the xanthophyll-binding protein DR_2577 (SlpA) and at least five more accessory subunits (25). This complex has been functionally and biochemically characterized over the last decade (3, 4, 12, 23, 24, 25, 26). Recently, the structure of its main subunit, the protein DR_2577, was partially solved at close-to-near atomic resolution (27) providing astonishing details in its organization, hence complementing our understanding of its functional traits. The SDBC appeared to be composed of three main regions: (i) a massive β-barrel organization structuring the pores' region, (ii) a β-sandwich organization characterizing the collar region, and (iii) a trimeric coiled coil for the stalk region. While the β-barrel was recently assigned and modeled with the protein DR_2577 (27), the other two regions remained to be solved. Here, using cryo-EM data, to which a local refinement strategy has been applied, we provide the structural details of the stalk and the collar regions. The latter is assigned as the protein DR_0644, a Cu-only superoxide dismutase (SOD), which was also functionally assayed and localized *in situ* by cryo-electron crystallography. Results provide primary insights into the strong resistance of *D. radiodurans* to electromagnetic stress. The SDBC is shown to be both a photoprotective system, through the xanthophyll-binding subunit DR_2577, and a chemoprotective one, by the SOD activity of the subunit DR_0644. Noteworthy, results provide a new view on the cell envelope/S-layer relationship in this bacterium, likely extendible to other species carrying porous S-layers. Remarkably, assemblies like the SDBC may have represented important templates for the evolution of early photosynthetic systems in which photochemical and electrochemical events are required to coexist (28, 29, 30, 31, 32, 33, 34). Finally, the known S-layer properties of self-assembling and transport, together with the photoprotective and chemoprotective ones here shown, raise this system as an ideal template for building new biomaterials exploitable in nanotechnology and biomedicine.

## Results

### The SDBC's collar region is a trimeric Cu-only SOD

**Figure 1:**
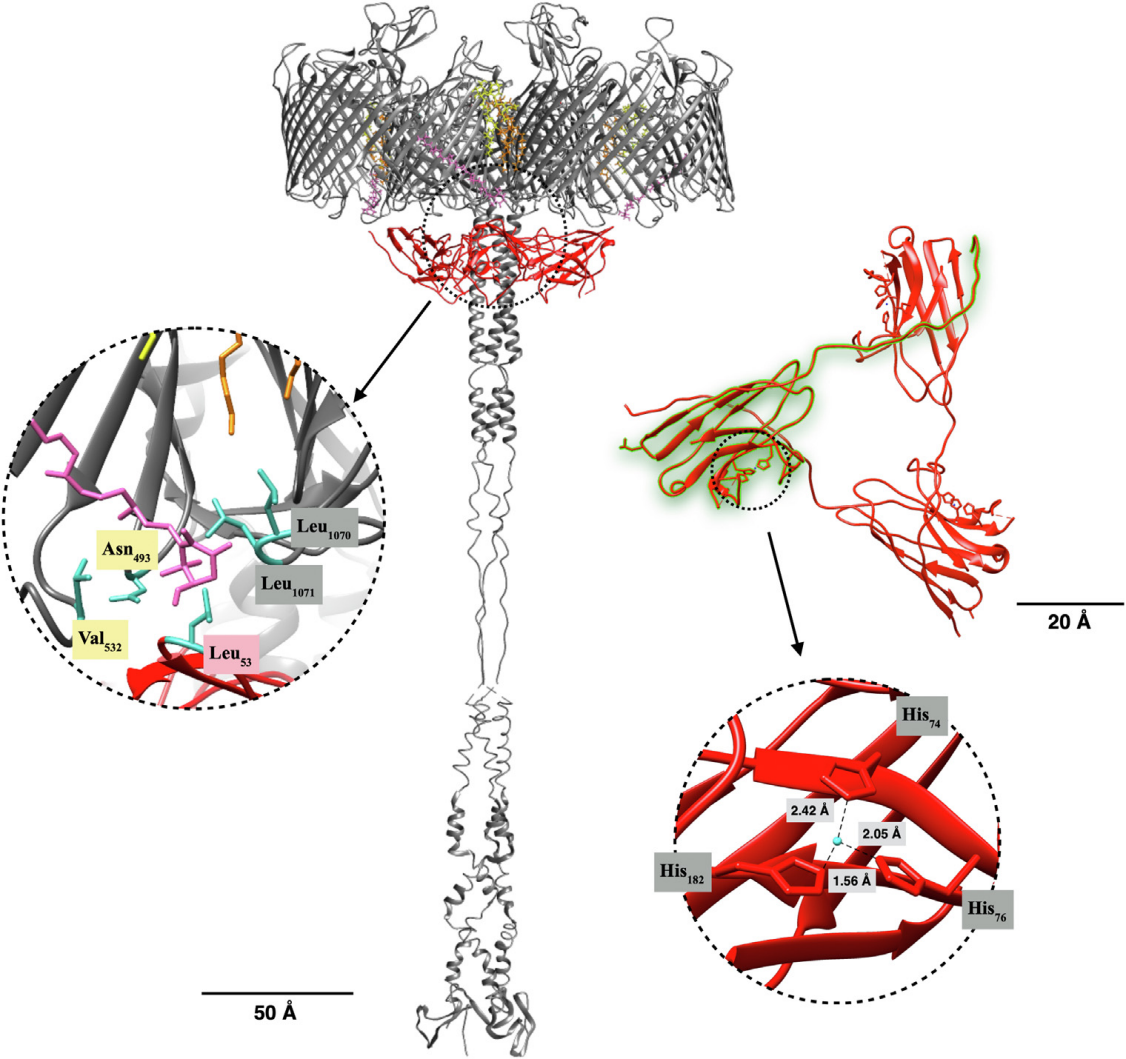
**Structure of the SDBC collar and DR_0644 model.** Overview of the SDBC model (*gray*), with highlighted (*red*) the DR_0644 trimer defining the collar region. The *upper right* inset shows the DR_0644 trimer observed from the periplasmic side evidencing its typical β-sandwich organization; one monomer is highlighted in *green* to *underline* the linking region. The *bottom left* inset shows a detail of the active site with the Cu-binding region coordinated by His_74_, His_76_, and His_182_, as typical for Cu-only SODs. In both insets, the Cu atoms are depicted in *blue*. The *left* inset shows a detail of the deinoxanthin-binding region. The scales indicate 50 Å and 20 Å, respectively.

The collar region of the SDBC was solved by cryo-EM at ∼2.5 Å resolution and found to consist of a single protein complex organized as a trimer (Fig. 1). This region was modeled as the uncharacterized protein DR_0644 (Uniprot entry Q9RWM2), previously reported to be present in the cell envelope, associated with the SDBC (22, 23, 24, 25), and here further characterized as a Cu-only SOD (35, 36, 37, 38). The protein sequence was modeled assigning 120 residues, missing a central threonine-rich region between the residues 90 and 137, which could be either disordered or truncated with respect to its ORF. Two short and disordered parts of the sequence, flanking the cleaved region upstream (residues 80–89) and downstream (residues 138–141), as well as a small N-terminal region interacting with the xanthophyll carotenoid deinoxanthin, remained unassigned (Leu_6_-Ala_19_; Fig. S1). The DR_0644 presents a typical β-sandwich motif, with six opposing antiparallel β-sheets, creating a structure with a width of ∼34 Å and a length of ∼85 Å (Fig. 1). The three monomers assemble into a trimer *via* a linking region of 17 residues at the N-terminal side (Gly_20_ – Pro_36_; Fig. S1). The trimer surrounds the coiled-coil region of the SDBC, and each DR_0644 monomer is localized under the interface between two DR_2577 pores where the specific cofactors, a deinoxanthin molecule and two characteristic phosphoglycolipids, are localized (Fig. 1). In particular, the Leu_53_ of each DR_0644 monomer appears to be in close proximity with the deinoxanthin molecule of each DR_2577 monomer (Fig. 1). As a result, each DR_0644 monomer interacts with the interface of two DR_2577 pores through their respective carotenoids (Fig. 1), *via* the N-terminal region in one interface (Leu_6_- Ala_19_; Fig. S1) and the Leu_53_ in the other. Bioinformatic analysis by basic local alignment (39) indicated the DR_0644 to be a SOD while metal ion-binding site prediction and docking studies (MIB) (40) allowed us to localize a single Cu-binding site for each SOD monomer. The Cu-binding site involves three characteristic His residues (His_74_, His_76_, and His_182_; Fig. 1), indicating DR_0644 to be a Cu-only SOD, a group of soluble enzymes typical for the periplasm and extracellular compartments (41, 42, 43, 44, 45) and here unusually found to be part of a cell envelope complex, the SDBC. Coordination and distances for the assigned binding site supported the presence of Cu^2+^ (Table S1).

### *In situ* analysis of the cell envelope confirms the SOD trimer to be localized in the periplasmic side

**Figure 2:**
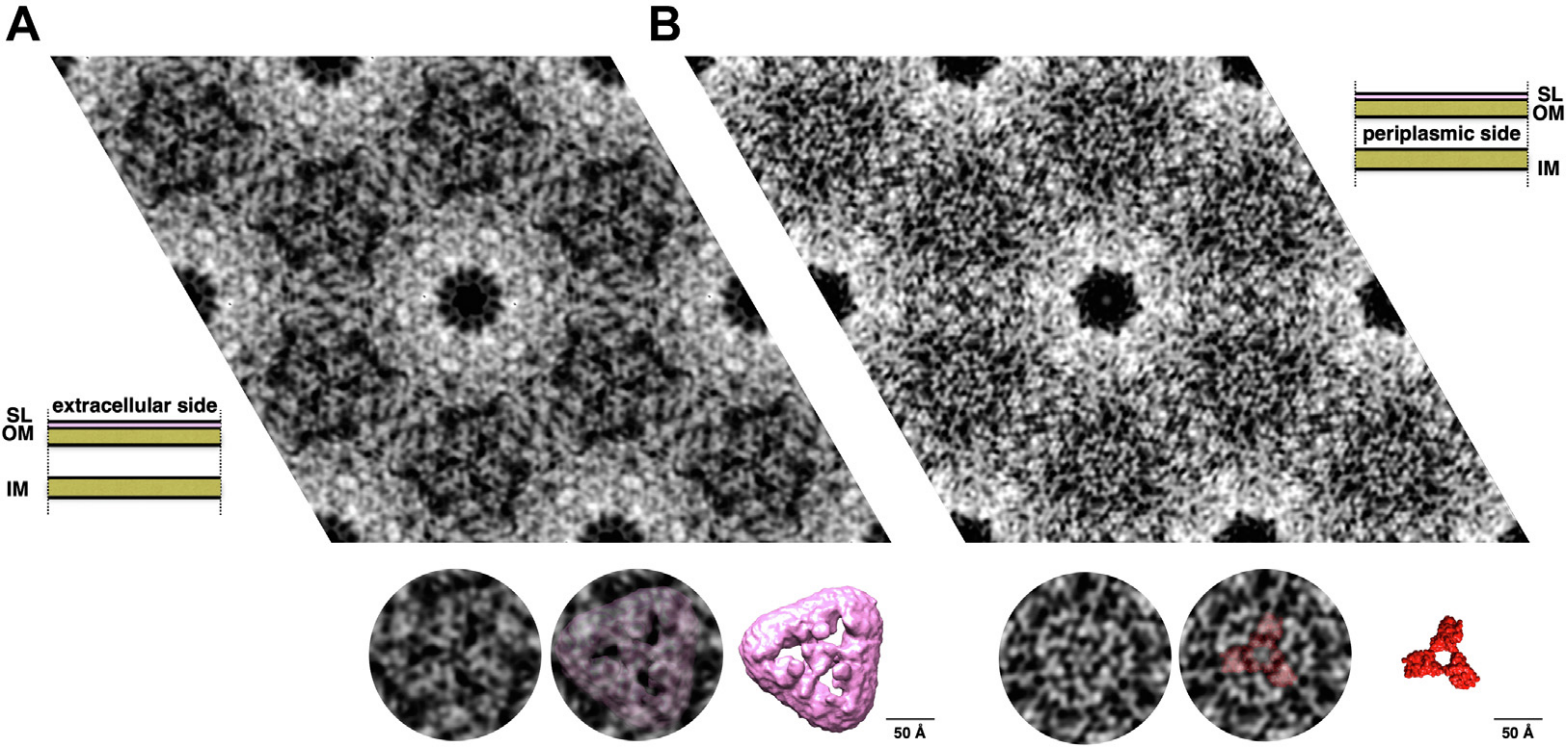
***In situ* localization of the SOD trimer.** Projection maps of the cell envelope with imposed p6 symmetry at 4.5 Å resolution were obtained from (*A*) six averaged images with dominant *top* views (extracellular face, as shown in the schematic representation of the cell envelope on the *bottom left*) and (*B*) six averaged images with dominant *bottom* views (cytosolic face, as shown in the schematic representation of the cell envelope on the *top right*). In the round insets below the maps, it is shown the DR_2577 top view (*center left*) and the DR_0644 *bottom* view (*center right*), respectively, fitted in the map. The remaining insets show a detail of the two maps and of the convoluted particles. From the fitting of the *bottom* view, it is evident that the collar region can be only partially explained by the contribution of the DR_0644 trimer. For both insets, the atomic models where convoluted into a 4.5 Å particle. The scale indicates 50 Å.

The SOD trimers, in the previous paragraph identified as the collar region of the SDBC, were localized *in situ* in intact cell envelope patches visualized by cryo-electron crystallography analysis. In *D. radiodurans*, the crystalline organization is extended to the whole cell envelope thickness (∼200 Å). For this reason, the diffraction analysis was attempted on the two main orientations of the cell envelope patches, which are the S-layer and the cytosolic sides, respectively. Due to the thickness, the 2D map of the first orientation carried dominant information of the S-layer side and consisted of a detailed 2D map of its components as shown previously (Fig. 2*A*) (20). In the second case, the main information is given by the highly packed regions at the periplasmic level, allowing seeing in great detail the periplasmic side and the less densely packed layers below (Fig. 2*B*). Importantly, the latter orientation allowed to visualize the SOD trimer *in situ*. The SDBC and the SOD structures were fitted in the 2D maps by convoluting the models into a particle at 4.5 Å, which is the in-plane resolution of the 2D map (Fig. 2 insets). This procedure also showed the presence of extra regions likely related to other SDBC subunits anchored to the complex and interacting with the SOD and the stalk.

### The stalk region is a noncanonical extended α-β coiled coil

**Figure 3:**
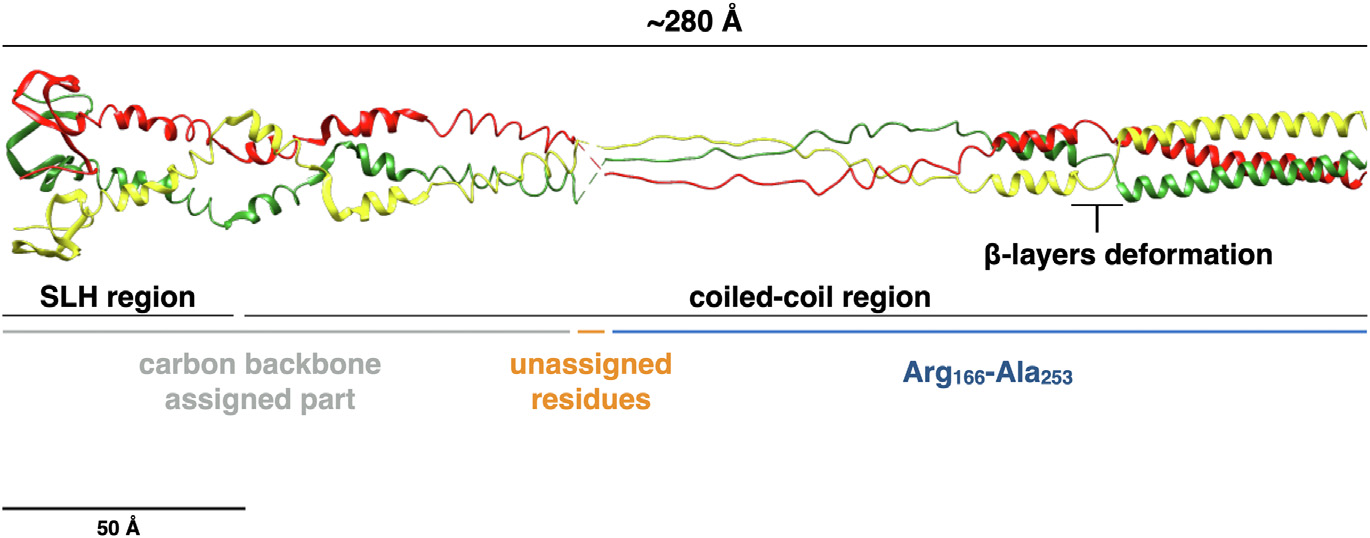
**Detail of the resolved SDBC stalk region.** An overview of the stalk region shows it as composed of an extended α-β coiled-coil region and the SLH region, covering the N-terminal part of the protein DR_2577. The stalk region appears to be extended for ∼280 Å, indicating a direct interaction with the inner membrane by the SLH domain. A detail of the assigned discontinuity (Val_213_-Ala_216_) resulting in local β-layers deformations is also indicated. In *blue*, it is shown the Arg_166-_Ala_253_ assigned region; in *orange*, it is shown the unassigned region, and in *light gray*, it is shown the N-terminal region for which the only carbon backbone could be assigned. The scale indicates 50 Å.

Previous structural studies on the SDBC did not allow resolving the first 218 residues at the N terminus due to a very long and dynamic coiled-coil stalk region (27). To overcome this problem, the cryo-EM data for this region were processed *de novo* using a local refinement approach on the stalk. This analysis allowed visualizing and modeling a large part of the unassigned coiled coil (residues 217–166) (Fig. 3), while downstream to the residue 166, after an interruption of 5 to 10 residues, it was possible to solve only the carbon backbone and reveal the SLH domain, which appeared to be organized into a glomerular structure (Fig. 3). The lower resolution of this region is most likely caused by dynamics within its parts and that might bring to local disorder while maintaining the whole general shape. The first part of the stalk region is a left-handed supercoil with a parallel orientation of the three chains and a periodicity of seven residues over two helical turns (heptads - 7/2). This trimeric coiled coil is also characterized by the presence of noncanonical motifs, of which only one was assigned (Val_216_-Ala_213_), resulting in local β-layers deformations forming the so-called α-β coiled coil (46) (Fig. 3).

Altogether, the stalk region is extended for ∼280 Å, making the total length of the SDBC ∼300 to 330 Å. The present findings confirm previous reports and indicate the extension of the stalk into the periplasm reaching and interacting with the inner membrane through its SLH domain (19, 20, 25, 27).

### The SDBC quenches UVC photons and ROS

**Figure 4:**
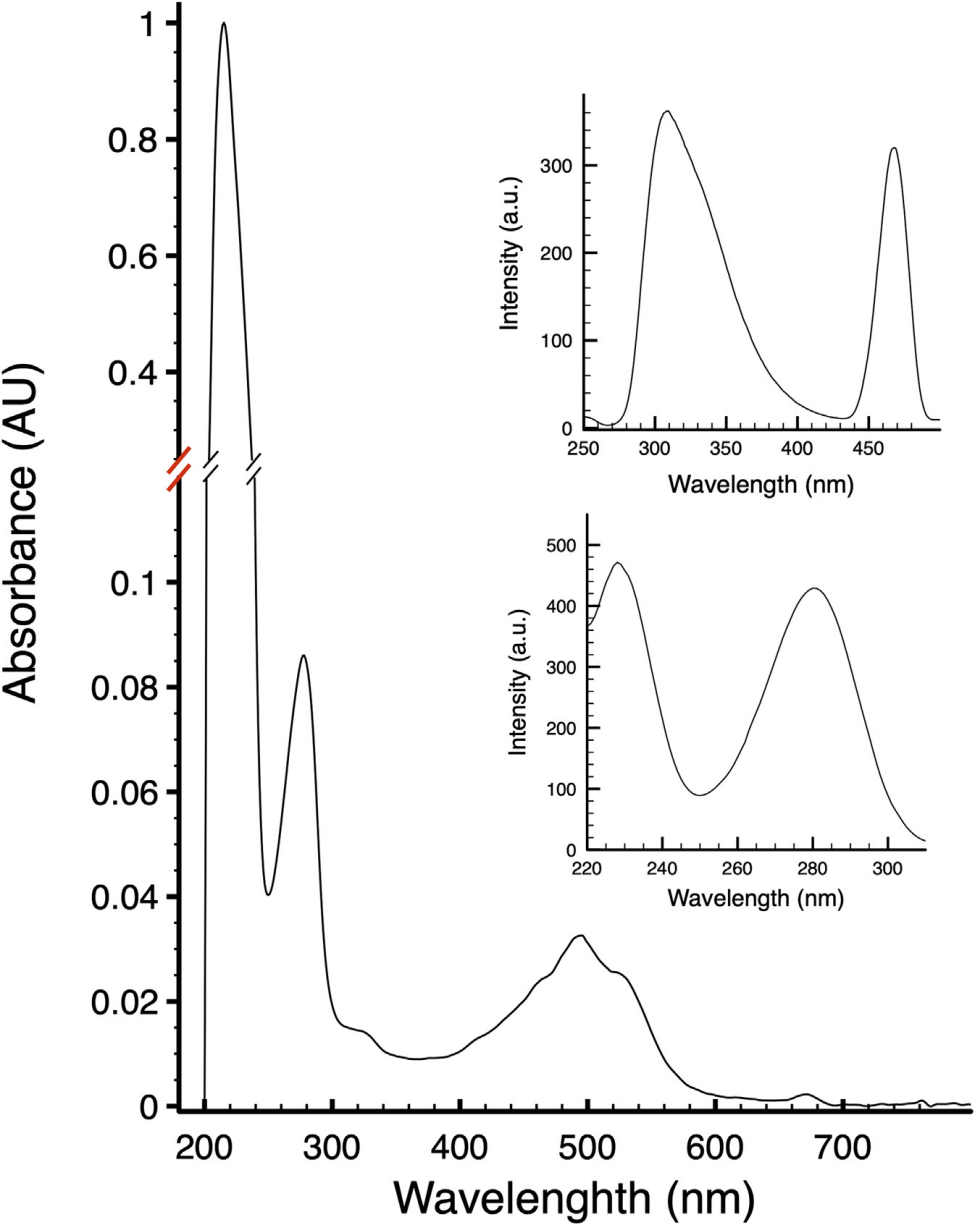
**SDBC spectroscopical properties.** Absorption spectrum of SDBC samples showing its typical absorption bands in the UV, due to aromatic amino acids, and in the visible, due to the xanthophyll carotenoid deinoxanthin. The insets on the *right* show a typical SDBC emission spectrum (*top*) and excitation spectrum (*bottom*) with 232 nm excitation and 325 nm emission, respectively.

The SDBC has been previously shown to have transport properties (20, 25, 27). Here, the quenching properties to protect against UV photons and ROS were assayed. The absorption spectrum of the SDBC shows the characteristic polyene signature of carotenoids in the visible, due to the xanthophyll-carotenoid deinoxanthin (Fig. 4). The main UV bands primarily result from the unusually high percentage of aromatic amino acid residues in the DR_2577 (∼9.8%) (3), the main subunit of the SDBC (25, 27) (Fig. 4).When characterized by fluorescence spectroscopy, the complex shows a clear tendency to absorb in the UVC-UVB and emit at longer wavelengths in the UVA-visible (Fig. 4 insets), indicating it as an important channel for energy dissipation.

In the second group of functional experiments, we assayed the SOD activity of the SDBC by using the rates of pyrogallol auto-oxidation as an indication of ROS superoxide ion (O_2_^−^) scavenging activity in the presence or absence of the SDBC (Table 1). The auto-oxidation rates of pyrogallol in presence of the SDBC at 1 min decreased of ∼85% with respect to the controls (as estimated by the values at 1 min in Table 1), indicating the activity of the SOD and allowing to quantify it as equivalent to ∼756 U/ml.

**Table 1:** Pyrogallol auto-oxidation assay on SDBC samples

Time	−SDBC	+SDBC	Antioxidant effect^a^
Minutes	Δ absorbance (AU)^b^		(%)
0	0	0	0
1	0.950 ± 0.131	0.139 ± 0.027	85.40
2	0.923 ± 0.132	0.173 ± 0.008	81.30
3	0.813 ± 0.055	0.157 ± 0.014	80.66
4	0.663 ± 0.032	0.133 ± 0.023	79.90
5	0.563 ± 0.055	0.107 ± 0.020	80.95

The rates of pyrogallol auto-oxidation were used as an indication of the ROS superoxide ion (O_2_^−^) scavenging activity of the SDBC and therefore of its Cu-only SOD subunit. The data show that in presence of the SDBC, the auto-oxidation at 1 min decreased by ∼85% compared to controls. This antioxidant activity was monitored for 5 min showing a similar effect.

a SDBC antioxidant effect expressed as rate (%) of pyrogallol protection with respect to the control (−SDBC); each value is the mean of three independent measurements.

b Difference of absorbance between measurements performed at a distance of 1 minute.

## Discussion

The SDBC is one of the main components in the cell envelope/S-layer of *D. radiodurans* (19, 20, 22, 25, 27). This assembly is characterized by the main core, the protein DR_2577 (or SlpA), and other loosely bound subunits (25). The atomic structure of the SDBC core was recently described showing its β-barrel organization consisting of a massive three-pores region inclusive of its ligands and cofactors (the xanthophyll carotenoid deinoxanthin, two exclusive phosphoglycolipids, the metals cations Cu^2+^ and Fe^2+^) (27). In that structure, the other SDBC regions, the stalk and the collar, remained partially or fully unresolved (27). Here, we improved the resolution of these regions by processing the SDBC particles with a local refinement strategy. This procedure allowed modeling the unassigned parts of the protein DR_2577 into a ∼280 Å long α-β coiled coil terminating with the SLH domain, providing implicit evidence for its interaction with the inner membrane. Furthermore, the collar region was also modeled and identified as the protein DR_0644, confirming previous characterizations by mass spectrometry (22, 25) and a previous model where the only carbon backbone was partially assigned (27). This protein was found here to be a Cu-only SOD important in scavenging one of the most dangerous and long-lived ROS, the superoxide ion (O_2_^−^). The lower resolution of the collar allowed to identify and assign the Cu-binding domain but not to localize the densities for the Cu ions, which were predicted with high confidence by different bioinformatic means (Table S1). The characteristic active site with the three coordinated histidines (Fig. 1) structurally confirmed the Cu-only SOD identity of this protein, corroborating the function of the collar region. While the internal dynamics of the Cu-only SOD are the most likely cause for the lower local resolution of the collar, the possibility that this could be also due to a partial movement of the collar along the periplasmic region of the stalk cannot be excluded. Importantly, high-resolution 2D maps obtained by cryo-electron crystallography allowed the *in-situ* localization of the collar on the periplasmic side of the SDBC (Fig. 2). Finally, the SDBC photoprotective role was further investigated by absorption/fluorescence spectroscopy (Fig. 4) and the SOD activity was assayed (Table 1).

Considering the nonselective transport properties of the SDBC and its regular organization around the entire cell surface (19, 20, 27), the ROS-scavenging ability of the collar region could efficiently and isotropically protect the bacterium from potentially adverse environmental conditions. This is in agreement with *D. radiodurans* being an extremophile known for its ability to cope with electromagnetic stress. This trait was most likely gained before the advent of oxygenic photosynthesis and the great oxygenation, when the absence of the atmospheric ozone filter allowed this radiation to reach the Earth surface (47, 48). Under these conditions, even if life started in water (49) and UV radiation could be efficiently shielded by this media, bacteria evolved several efficient mechanisms of photoprotection, by quenching the incoming UV photons, and chemoprotection, by scavenging the resulting ROS derivatives from photosensitizing reactions to water.

In this context, the SDBC of *D. radiodurans* represents an element of the cell envelope's functionalization carrying active protection properties aimed at preventing and repairing occurring damages. The identification of the SOD subunit implies that the SDBC exploits the photoprotection given by its core, the protein DR_2577, to prevent damages that are otherwise repaired by its SOD chemoprotective component, the protein DR_0644. Similar systems might have been essential for the evolution of primordial photosynthetic systems that had to combine photoprotection and chemoprotection with energy production (50, 51). Photo/chemoprotective systems such as the SDBC, carrying both a light-harvesting system (mediated by light-absorbing amino acid residues and carotenoids) and an oxidoreductase activity (sustained by the reactions characteristic for SODs), might have represented a functional template for combining energetic needs with protection mechanisms. This observation, and in particular the connection between SODs and the photosynthetic reactions centers, is extensively reported (28, 29, 30, 33), so that the reported findings could represent an ideal system to investigate and possibly corroborate this hypothesis. What could be the reasons for maintaining an anachronistic structure such as the SDBC? While the hierarchical organization of the cell envelope/S-layer might provide a reason related to the stability of the cell, also its transport properties might represent an important evolutive reason for keeping it in place. However, the high energetic costs required for expressing, placing, and maintaining this structure might suggest some more important existing reasons so that also possible productive processes of photoconversion cannot be excluded and must be further investigated. The present findings provide important insights to understand evolutive aspects crucial for the origin and sustain of life. The selective pressure played on evolution by environmental stressors of physical or chemical origin might represent the primary reason for protective molecular machines such as the SDBC that could be suitable as evolutive templates for productive aims. Considering the peculiar properties of S-layer proteins and in particular their high stability in harsh conditions and self-assembling into crystalline bidimensional surfaces, the SDBC has the potential of an exploitable “brick” for building new biomaterials and producing devices for nanotechnological and biomedical applications.

## Experimental procedures

### Cell culturing and SDBC purification

*D. radiodurans* (strain R1; ATCC 13939) was grown in Tryptone Glucose Yeast extract broth (TGY) at 30 °C for 24 h as described in (22). The cell envelope patches and the SDBC purifications were performed according to (19, 20, 25, 27) with no modifications. In this study, chromatography columns were subjected to the ReGenFix procedure (https://www.regenfix.eu/) for regeneration and calibration prior to use.

### Cryo-EM

Samples preparation and data acquisition were done as reported in (20, 27) with no modifications. Briefly, the SDBC sample (3.5 μl at a concentration of 5 mg/ml) was applied to freshly glow-discharged transmission electron microscopy grids (Cu 300 mesh, R1.2/1.3 - Protochips) and vitrified into liquid ethane (4 °C, 100% rel. humidity, 30 s waiting time, 3 s blotting time) using a Vitrobot Mark IV (FEI). Grids were subsequently mounted into the Autogrid cartridges. The screening prior to data acquisition was done using a FEI Talos Arctica operating at 200 kV (ThermoFisher). Data were collected using the SerialEM software (https://bio3d.colorado.edu/SerialEM/) (52) at the calibrated pixel size of 0.818 Å/px (nominal magnification of 1,650,00×) on a FEI Titan Krios operating at 300 kV, aligned for fringe-free imaging and equipped with Cs-corrector (cs 2.7 mm), a Quantum GIF energy filter (slit width set to 10 eV), and a post-GIF K2 camera (Gatan). The data from a 5.0 s exposure were saved into 40 frames containing overall dose of 55 e/Å^2^. The dataset comprised 9594 movies in total. The image defocus was set to vary between −0.5 μm and −1 μm. The sample preparation and the data acquisition were both done at CEITEC, Brno, Czech Republic.

### Data analysis

Cryo-electron crystallography data analysis was performed according to (20). The cell envelope patches' orientation was discerned by using 2D maps obtained from single micrographs processed with no imposed symmetry (p1). Next, for each orientation, the best six images were merged and processed with a p6 symmetry imposed according to (20).

Single-particle data collection and analyses were performed according to (27) with no modifications. The SDBC maps with imposed C1 and C3 symmetry (2.88 Å resolution, EMD-14714, and 2.54 Å resolution, EMD-14715) were used to do a *de novo* modeling of the collar region (DR_0644) to assign the amino acid sequence and refinement. In detail, the assignment of the sequence and the initial modeling of the monomer were done on the nonsymmetrized map (C1, EMD-14714) while the trimer was subsequently modeled and refined on the symmetrized map (C3, EMD-14715), which, however, showed significant heterogeneities in this region, suggesting the presence of specific dynamics between monomers. Further data processing was performed by local refinement of two segments of the stalk, the region immediately below the collar and the SLH domain. In both cases, a C3 symmetry was imposed (maps at 3.5 Å resolution, EMD-15382, and 5.3 Å resolution, EMD-15384, respectively; composite map, EMD-15487), allowing to model the stalk region. Assignment and modeling were accomplished using Coot software (https://www2.mrc-lmb.cam.ac.uk/personal/pemsley/coot/) (53), and the resulting model was refined with the Phenix software (https://phenix-online.org/) (54). Model visualization and fittings were done using the Chimera software (https://www.cgl.ucsf.edu/chimera/) (55).

### SOD activity

Isolated SDBC samples (0.2 mg/ml) were used to assess the SOD activity by a spectrophotometric assay. For the blank, the solution A (60 mM Tris–HCl pH 8.2, 0.5 mM EDTA) was mixed with the solution B (4.5 mM pyrogallol, 10 mM HCl) in a ratio of 29/1, according to common enzymatic assay protocols (Creative Enzymes). For the test sample, the SDBC was added to the solution A in a ratio of 1/20, reaching a final protein concentration of 0.01 mg/ml in the total volume mix (solutions A + B). In both cases, after vortexing, the absorbance was measured at 325 nm at time zero and each minute for 5 min. The differences in absorbance between time zero and after 1 min was used as an indication of the rate of pyrogallol auto-oxidation, while the differences between the two samples at 1 min was used to estimate the SOD activity according to the following formula:

$$SOD\;activity\left(U/mL\right)=\frac{\frac{\Delta A_{325blank}-\Delta A_{325sample}}{\Delta A_{325blank}}\times 100\%}{50\%}\times 0.9\times \frac{1}{V}\times D$$

where U/ml is the SOD activity unit, ΔA_325 blank_ is the auto-oxidation rate in the blank, ΔA_325 sample_ is the auto-oxidation rate using the SDBC, V is the SDBC volume in milliliter, D is the dilution factor of the sample, and 0.9 is the total volume in milliliter of the reaction mixture. All measurements were performed at 18 °C and repeated in triplicates. Finally, the data were plotted using plot2 (https://apps.micw.org/apps/plot2/).

### Spectroscopy measurements

Absorption and fluorescence spectra of the SDBC were recorded at a protein concentration of 0.2 mg/ml. Absorption measurements were performed on a Pharmacia Biotech Ultrospec 4000 spectrophotometer at 18 °C in the range of 200 to 750 nm with an optical path length of 1 cm and a band-pass of 2 nm. Fluorescence measurements were accomplished on a F-2500 FL Spectrophotometer (Hitachi) at 232 nm excitation in emission mode and 325 nm emission in excitation mode. Measurements were done at 18 °C with a scan speed of 1500 nm/min and a slit width of 10 nm. All spectra were recorded on an absorption Ultra Micro quartz cell with 10 mm light path (Hellma Analytics).

### Data and materials availability

The coordinates file and other relevant information about data acquisition and processing have been deposited in the Electron Microscopy Data Bank (EMDB; https://www.ebi.ac.uk/emdb/): SOD trimer, PDB-8ACA; SDBC assembled complex (DR_2577 and DR_0644), PDB-8ACQ and PDB-8AGD; SDBC map with details of the coiled-coil region, EMD-15382; SDBC map with details of the N-terminal region of the coiled-coil region and SLH domain, EMD-15384; SDBC assembled complex (full DR_2577 and DR_0644), composite map, EMD-15487. All data will be made publicly available upon acceptance of the manuscript.

## Supporting information

This article contains supporting information.

## Conflict of interests

The authors declare that they have no conflicts of interest with the contents of this article.
