# Supplementary material for: The SDBC is active in quenching oxidative conditions and bridges the cell envelope layers in Deinococcus radiodurans
Source: J Biol Chem. 2022 Dec 9;299(1):102784. doi: 10.1016/j.jbc.2022.102784 (PMC9823218; doi:10.1016/j.jbc.2022.102784)
Supplement: Supplemental Figure S1 and Table S1 [file mmc1.doc]

A S-layer complex is an active shield against extreme conditions

in *Deinococcus radiodurans*

Domenica Farci, André T. Graça, Luca Iesu, Daniele de Sanctis, Dario Piano

**Supplementary material**

**Supplementary** Figure 1.

Assigned and missing parts of the DR_0644 sequence. The missing first 5 residues on both N-terminal and C-terminal sides are indicated in red. The cleaved central part rich in threonines, consisting of 47 residues (90-137), is also indicated in red. The disordered and difficult-to-assign regions, flanking the threonine-rich region (residues 80-89 and 138-141) and upstream the N-terminal region (residues 6-19), are indicated in blue, while the remaining modeled part is shown in green.


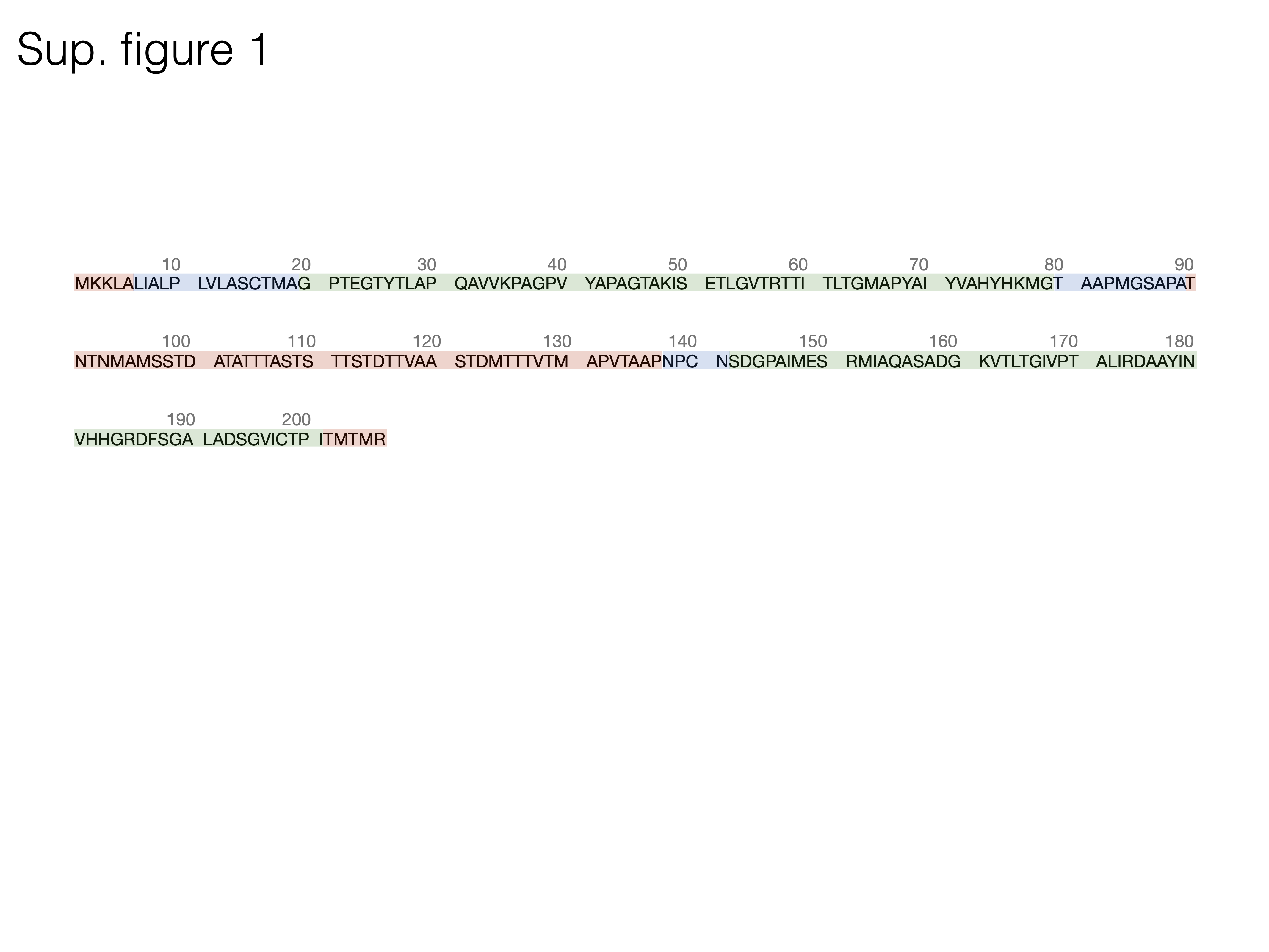


**Supplementary Table 1 –** Metal-binding assignment evaluation according to the CMM validation server.

| **ID** | **Metal** | **Occupancy** | **Ligands** | **Valence** | **Geometry** | **gRMSD (°)** | **Vacancy** | **Bidentate** | **Alternative metal** |
| --- | --- | --- | --- | --- | --- | --- | --- | --- | --- |
| **SOD (IA)** | Cu | 1 | N2 | 2.2 | Square planar | 6.4° | 0.5 | 0 | n.a.* |
| **SOD (IB)** | Cu | 1 | N3 | 2.1 | Tetrahedral | 20.6° | 0.25 | 0 | n.a.* |
| **SOD (IC)** | Cu | 1 | N3 | 1.5 | Tetrahedral | 15.5° | 0.25 | 0 | n.a.* |

***n.a.** = not applicable.
